# Effects of Concomitant Ovariohysterectomy During Elective Caesarean Section in Bitches: A Prospective Clinical Study

**DOI:** 10.1111/rda.70334

**Published:** 2026-11-05

**Authors:** Juliana Dantas Bento, Mariana Semião Francisco Talib, Marcelo Faustino, Álvaro de Miranda Alves, Gisele Almeida Lima da Veiga

**Affiliations:** ^1^ Department of Animal Reproduction, School of Veterinary Medicine and Animal Science University of São Paulo São Paulo Brazil

**Keywords:** canine reproduction, maternal behaviour, neonatal care, obstetrics, puerperium, surgical sterilization

## Abstract

The association of ovariohysterectomy with caesarean section in bitches remains controversial due to concerns regarding surgical time, haemorrhage, maternal recovery, lactation, and neonatal care. This prospective study evaluated intraoperative findings, postoperative recovery, and puerperal outcomes in bitches undergoing elective caesarean section with or without concomitant ovariohysterectomy. Twenty‐five pregnant bitches undergoing elective caesarean section were prospectively enrolled and classified according to the surgical procedure performed into a Caesarean Group (CG, *n* = 15) and a Caesarean plus Ovariohysterectomy Group (COHG, *n* = 10). Group assignment was not randomized and was determined by owner consent and clinical and reproductive management considerations. Clinical, surgical, and postpartum variables were assessed from the preoperative period until 30 days postpartum. No statistically significant differences were detected between groups regarding anaesthetic time, surgical time, total weight of blood‐contaminated surgical waste, postoperative recovery, lactation, neonatal viability, or wound healing. Vital parameters remained within physiological limits throughout the puerperal period in both groups, although heart rate differed significantly between groups at 30 days postpartum. One bitch in the COHG group was not available for behavioural and lactation assessments. Maternal rejection was observed in 13.3% (2/15) of bitches in the CG and 55.6% (5/9) in the COHG group, although the difference did not reach statistical significance (*p* = 0.061). No major maternal or neonatal complications were observed during the study period. However, the limited sample size, particularly in the COHG group, warrants cautious interpretation of these findings and limits the ability to exclude clinically relevant differences between groups. The possible influence of concomitant ovariohysterectomy on maternal behaviour warrants further investigation.

## 1 Introduction

Caesarean section is commonly performed in canine obstetrics, particularly in breeds predisposed to dystocia and in high‐risk pregnancies. Recent studies have demonstrated an increasing frequency of elective and emergency caesarean sections in bitches, especially among brachycephalic breeds and females with a previous history of dystocia or caesarean delivery (Schrank et al. 2022). Elective caesarean section is generally planned before the onset of clinical dystocia in selected pregnancies considered at increased risk for difficult parturition, whereas emergency caesarean section is performed when dystocia or maternal or foetal compromise requires urgent intervention. These two clinical settings may differ in maternal and foetal characteristics and in the circumstances surrounding surgery and may consequently influence neonatal and postoperative outcomes. In bitches, emergency caesarean section has been associated with higher perinatal mortality than scheduled procedures (Rodríguez et al. 2024), and neonatal survival to hospital discharge has also been reported to be lower following emergency compared with elective caesarean section (Adams et al. 2022). Therefore, the present study was specifically designed to evaluate bitches undergoing elective caesarean section under planned clinical conditions.

In some clinical situations, ovariohysterectomy is performed concurrently with caesarean section as a definitive contraceptive procedure. Potential advantages include prevention of future dystocia, elimination of subsequent oestrous cycles, and avoidance of an additional anaesthetic and surgical procedure. However, the safety of combining both procedures remains controversial in small animal reproduction practices.

The main concerns associated with ovariohysterectomy during caesarean section include increased surgical time, intraoperative haemorrhage, postoperative pain, impaired maternal behaviour, decreased lactation, possible perioperative haematological changes associated with surgical blood loss, and possible adverse neonatal outcomes. Blood loss during caesarean section may contribute to transient reductions in packed cell volume (PCV) and red blood cell (RBC) counts, making postoperative haematological assessment relevant when evaluating maternal perioperative outcomes (De Cramer et al. 2016). Despite these concerns, clinical evidence evaluating maternal and neonatal consequences of concurrent ovariohysterectomy remains limited.

Recently, Guest et al. (2023) retrospectively evaluated 125 bitches undergoing caesarean section with or without concurrent ovariohysterectomy and reported no increase in perioperative complications, maternal mortality, or decreased mothering ability associated with the combined procedure. However, this retrospective study primarily addressed perioperative outcomes and did not prospectively characterise the short‐term maternal recovery, maternal behaviour, lactation, and early puerperal adaptation following concomitant ovariohysterectomy. In addition, prospective longitudinal evaluations of these outcomes in bitches undergoing elective caesarean section remain scarce. Therefore, an important knowledge gap concerns the prospective assessment of maternal and neonatal outcomes during the immediate postoperative and subsequent puerperal period following the combined procedure.

The puerperal period in bitches is characterized by complex endocrine and behavioural adaptations associated with maternal care, lactation, and neonatal survival. Surgical sterilization immediately after foetal delivery could potentially influence maternal behaviour and postpartum adaptation, although evidence supporting this hypothesis is still insufficient.

Therefore, the aim of the present prospective study was to evaluate intraoperative findings, postoperative recovery, maternal behaviour, lactation, and puerperal outcomes in bitches undergoing elective caesarean section with or without concomitant ovariohysterectomy.

## 2 Materials and Methods

### 2.1 Ethical Approval

This study was approved by the Ethics Committee on the Use of Animals of the School of Veterinary Medicine and Animal Science, University of São Paulo, under protocol number 7693020924.

### 2.2 Animals and Experimental Design

Twenty‐five pregnant bitches referred for elective caesarean section at the Gynaecology and Obstetrics Service of the Department of Animal Reproduction, School of Veterinary Medicine and Animal Science, University of São Paulo, were prospectively enrolled in this study between September 2024 and August 2025. During the study period, 26 bitches were assessed for eligibility. One bitch was excluded because of aggressive behaviour that prevented adequate handling during the preoperative evaluation and subsequent postoperative assessments.

Animals were classified into two groups according to the surgical procedure performed. Group assignment was not randomized. Among bitches undergoing elective caesarean section, concomitant ovariohysterectomy was considered when future breeding was not desired, following discussion between the attending veterinarian and the owner. In two multiparous breeding bitches with a history of multiple previous pregnancies, the decision to perform concomitant ovariohysterectomy was additionally supported by intraoperative findings of extensive uterine adhesions and altered uterine positioning. The Caesarean Group (CG, *n* = 15) consisted of bitches undergoing elective caesarean section without surgical sterilization, whereas the Caesarean plus Ovariohysterectomy Group (COHG, *n* = 10) included bitches submitted to elective caesarean section associated with ovariohysterectomy.

Breed, age, body weight, reproductive history, previous caesarean section, and number of previous parturitions were recorded before surgery. Age and number of previous parturitions are summarized in Table 1. All animals underwent physical and obstetrical examinations before inclusion in the study. Preoperative, intraoperative, postoperative, and puerperal evaluations were performed in all animals from admission until 30 days postpartum.

**TABLE 1:** Preoperative and intraoperative variables in bitches undergoing elective caesarean section with or without concomitant ovariohysterectomy.

Variable	CG	COHG	*p*
Number of previous parturitions, median (IQR)	1 (0–1)	0 (0–1)	0.6096
Age (years)	2.27 ± 0.23	3.70 ± 0.42	0.0036
Surgery time (min)	67.73 ± 3.13	80.20 ± 6.01	0.0563
Anaesthesia time (min)	116.60 ± 6.31	113.40 ± 7.55	0.7491
Anaesthetic induction time 1^a^	36.20 ± 2.86	28.60 ± 2.52	0.0747
Anaesthetic induction time 2^b^	53.67 ± 4.44	44.00 ± 4.25	0.1484
C‐section time (min)	34.20 ± 2.25	40.6 ± 5.75	0.3212
Total weight of blood‐contaminated surgical waste (kg)	1.47 ± 0.20	1.38 ± 0.28	0.7706
Number of neonates delivered	6.07 ± 0.61	6.40 ± 1.11	0.796
Preoperative heart rate (bpm)	113.20 ± 4.33	126.22 ± 9.48	0.1695
Preoperative respiratory rate (breaths/min)	34.29 ± 1.91	28.00 ± 4.28	0.1342
Preoperative rectal temperature (°C)	37.09 ± 0.17	37.19 ± 0.22	0.7321
Capillary refill time	1.79 ± 0.11	1.89 ± 0.11	0.571

*Note:* Data are presented as mean ± standard error of the mean (SEM), except for number of previous parturitions, which is presented as median (interquartile range [IQR]).

^a^ Anaesthetic induction time 1—Anaesthetic induction time from anaesthetic induction to the delivery of the first neonate.

^b^ Anaesthetic induction time 2—Anaesthetic induction time from anaesthetic induction to the delivery of the last neonate.

### 2.3 Inclusion and Exclusion Criteria

Only clinically healthy pregnant bitches undergoing elective caesarean section at term were included in the study. Gestational age was estimated based on breeding history, ultrasonographic foetal development, and serum progesterone concentrations. Elective caesarean section was indicated according to the institutional obstetrical protocol based on maternal history, breed predisposition to dystocia (particularly brachycephalic breeds), previous caesarean section, singleton pregnancy, suspicion of foetal oversize, clinical examination, assessment of foetal maturity and other conditions associated with an increased risk of dystocia. Concurrent ovariohysterectomy was performed after owner consent when future breeding was not desired. In two multiparous breeding bitches with a history of multiple previous pregnancies, the decision to perform concurrent ovariohysterectomy was further supported by intraoperative findings of extensive uterine adhesions and marked distortion of the uterine anatomy.

Animals presenting systemic disease, severe foetal distress, obstructive dystocia requiring emergency intervention, septic conditions, severe haemorrhage before surgery, or perioperative complications unrelated to the experimental procedures were excluded. Bitches that had received any medication or nutritional supplementation during the preoperative period were excluded from the study, regardless of pharmacological class or type of supplementation, in order to minimize potential confounding effects on maternal behaviour, neonatal vitality, and lactation. This criterion included, but was not limited to, analgesics, opioids, α2‐adrenergic agonists, sedatives, corticosteroids, hormonal or lactogenic agents, vitamins, minerals, and other nutritional supplements that could potentially influence the outcomes assessed.

### 2.4 Anaesthetic Protocol

All animals were subjected to the same anaesthetic protocol. Premedication consisted of dexmedetomidine (1 μg/kg IV). Anaesthetic induction was performed using propofol (3–5 mg/kg IV), followed by lumbosacral or lumbar epidural anaesthesia with morphine (0.1 mg/kg) and lidocaine (4 mg/kg). In animals undergoing lumbosacral epidural anaesthesia, a final volume of 0.36 mL/kg was administered. The induction to extraction interval was defined as the time elapsed between administration of the induction agent and extraction of the first neonate and was recorded for each bitch. The epidural block was performed immediately after anaesthetic induction, followed by positioning of the bitch in dorsal recumbency and immediate preparation for celiotomy and hysterotomy. The specific duration from completion of the epidural block to extraction of the first neonate was not recorded separately, as this interval comprised the immediate transition from completion of the epidural procedure to positioning and surgical access for foetal extraction. Anaesthesia was maintained with isoflurane in oxygen.

The anaesthetic protocol was selected based on its established use in canine caesarean section and the need to provide adequate maternal sedation and analgesia while minimizing potential effects on neonatal vitality. Dexmedetomidine was administered at a low dose (1 μg/kg IV) as premedication. Previous studies in bitches undergoing caesarean section have reported favourable maternal and neonatal outcomes with dexmedetomidine or medetomidine containing protocols (De Cramer et al. 2017; Groppetti et al. 2019). Although placental transfer of dexmedetomidine has been demonstrated, available evidence has not shown major adverse effects on neonatal vitality under the conditions and doses evaluated (Groppetti et al. 2019; Di Cesare et al. 2025). A recent systematic review further indicated that low preoperative doses of dexmedetomidine were not associated with significant reductions in puppy vitality, while emphasizing the heterogeneity and methodological limitations of the available evidence (Navarro‐Altuna et al. 2025). Therefore, the use of low dose dexmedetomidine in the present study was based on available clinical evidence, while recognizing that the effects of α2‐adrenergic agonists on neonatal adaptation remain incompletely characterized.

Maternal heart rate, respiratory rate, rectal temperature, capillary refill time, peripheral oxygen saturation, and arterial blood pressure were monitored throughout the anaesthetic procedure.

### 2.5 Surgical Procedures

All surgical procedures were performed by the same surgeon. Caesarean sections were performed through a ventral midline celiotomy. After uterine exposure, a longitudinal hysterotomy was consistently performed on the ventral aspect of the uterine body, close to the uterine bifurcation, allowing sequential foetal extraction. This standardized approach was used in all animals. Umbilical cord ligation and neonatal assistance were subsequently performed by a separate team. After all neonates had been extracted, the uterus was routinely sutured in two inverting layers using Cushing and Lembert suture patterns with poliglecaprone suture material. In animals allocated to the Caesarean plus Ovariohysterectomy Group (COHG), the uterine incision was temporarily occluded using atraumatic clamps, without suturing, and ovariohysterectomy was subsequently performed using a standard three‐clamp technique, in which three hemostatic clamps were sequentially applied to the ovarian pedicle and uterine body to facilitate secure ligation and transection of the tissues. Ovarian pedicles were ligated using non‐absorbable monofilament nylon suture material, which was left in situ following surgery as part of the routine surgical protocol. The uterine body was subsequently transfixated using the same suture material.

Maternal clinical evaluations were performed immediately before and after surgery. Heart rate (HR) and respiratory rate (RR) were assessed by thoracic auscultation using a stethoscope, rectal temperature (RT) was measured using a digital thermometer, and capillary refill time (CRT) was evaluated by digital pressure applied to the gingival mucosa. During surgery, maternal vital parameters were continuously monitored using a multiparameter monitor, including heart rate, respiratory rate, rectal temperature, arterial blood pressure (ABP), oxygen saturation (SpO_2_), end‐tidal carbon dioxide (EtCO_2_), and electrocardiography (ECG).

Celiorrhaphy was performed according to the anatomical planes. Muscular closure was performed using simple interrupted sutures with poliglecaprone suture material. Subcutaneous tissue was routinely apposed using poliglecaprone, and skin closure was performed using an intradermal suture pattern with the same material. Postoperative treatment was standardized in all animals and included cephalexin (30 mg/kg BID for 5 days), dipyrone (25 mg/kg TID for 3 days), and tramadol hydrochloride (2 mg/kg TID for 3 days). Postoperative antimicrobial treatment followed the institutional protocol in effect during the study period.

At the end of each surgical procedure, all materials collected for weighing were placed in a single disposal bag. These materials included placentas, blood‐soaked surgical sponges and gauzes, and other disposable materials generated during surgery. The total weight was recorded as an indirect intraoperative parameter for comparison between groups.

The following intraoperative variables were recorded throughout the surgical procedure: surgical time, anaesthetic time, interval between anaesthetic induction and foetal delivery, caesarean section duration, total weight of blood‐contaminated surgical waste, and maternal vital parameters.

### 2.6 Postoperative and Puerperal Evaluation

Postoperative and puerperal evaluations were performed during the first postoperative hours and subsequently at 24 h and 7, 15, and 30 days postpartum by the same investigator. Maternal survival and neonatal viability were monitored throughout the entire 30‐day postpartum follow‐up period. At each evaluation, maternal vital parameters, maternal clinical status, surgical wound healing, vulvar discharge, lactation, and neonatal viability were assessed. Neonatal vitality was objectively assessed using the Apgar scoring system described by Veronesi et al. (2009), with individual scores recorded immediately after birth for subsequent analysis. All immediate neonatal assessments and neonatal care were performed by a dedicated team of neonatologists, independently of the surgical team. Neonatal viability and survival were monitored throughout the 30‐day postpartum follow‐up period. Maternal vital parameters included heart rate (HR), respiratory rate (RR), rectal temperature (RT), capillary refill time (CRT), and mucous membrane colour. Maternal clinical status included level of alertness, appetite, water intake, defecation, urination, and the presence of abnormal behaviour. All bitches remained hospitalized in the maternity ward for 24 h after surgery. Hospital discharge was performed 24 h postpartum, provided that the bitch was clinically stable, with normal vital parameters, appropriate maternal behaviour, successful nursing, and no postoperative complications, and that the puppies were clinically healthy and viable. Vulvar discharge was assessed during each postoperative and puerperal evaluation by direct inspection of the vulva and recorded as present or absent. Because the ovariohysterectomy was performed cranial to the cervix, the cervical stump was preserved. Therefore, in bitches undergoing concomitant ovariohysterectomy, vulvar discharge referred to secretions originating from the cervical stump and vaginal rather than uterine lochia. Surgical wound healing was evaluated at each time point for signs of inflammation, seroma formation, edema, haemorrhage, and wound dehiscence. Lactation was assessed by manual expression of the mammary glands to confirm milk production and by observation of effective suckling by the puppies.

Maternal behaviour was assessed using predefined behavioural criteria rather than a validated quantitative scoring system. Assessments were performed by the same investigator at predefined time points: during the first postoperative hours, at 24 h postpartum, and subsequently at 7, 15, and 30 days postpartum. During the first postoperative hours and at 24 h postpartum, maternal behaviour was assessed during direct maternal‐neonatal interaction while the animals remained hospitalized in the maternity ward. Following hospital discharge, maternal behaviour was evaluated during scheduled follow‐up visits at 7, 15, and 30 days postpartum based on owner interviews and direct observation whenever possible. At each assessment, maternal acceptance and rejection were classified according to the predefined behavioural criteria described below. Maternal acceptance was evaluated based on the willingness to interact with the puppies, nursing behaviour, licking and grooming of the neonates, and stimulation of urination and defecation. Maternal rejection was defined as avoidance of the litter, refusal to nurse, attempts to bite the puppies, growling, or moving away from the neonates. Persistent maternal avoidance was defined as repeated avoidance of the litter during consecutive behavioural assessments throughout the 24‐h hospitalization period despite repeated opportunities for maternal interaction. No anxiolytic, tranquillising, or other pharmacological interventions were administered to modify maternal behaviour. The investigator performing the maternal behaviour assessments was not blinded to the surgical group, which may have introduced observer bias and represents a limitation of the study.

Lactation was assessed during the first postoperative hours and subsequently at 24 h and 7, 15, and 30 days postpartum. Milk production was evaluated by complete manual milking of all mammary glands every 15 min over a 2‐h period. The total volume of milk obtained during this period was measured and compared with the expected total milk volume, calculated based on the total body weight of the neonates in the litter. The expected total milk volume was estimated using half of the neonatal gastric capacity immediately after birth (approximately 2.5 mL per 100 g of body weight), according to the following formula (Menezes et al. 2027): ETV (mL) = TNW (g) × 2.5/100, where ETV is the expected total milk volume and TNW is the total neonatal body weight of the litter. Hypogalactia was diagnosed when the total milk volume obtained during the 2‐h assessment period was lower than the calculated expected total milk volume. Bitches diagnosed with hypogalactia received oxytocin (0.1 IU/kg, IM) every 24 h for a total of three doses as part of the standard postoperative clinical management. When hypogalactia persisted for more than 2 h, supplemental feeding was provided to the neonates according to the standardized protocol of the neonatology team. Lactation was reassessed during hospitalization and at subsequent postpartum evaluations. Because lactation normalized in all bitches during the postpartum follow‐up, only the findings from the immediate postoperative assessment were included in the statistical analysis. The surgical wound was evaluated during the first postoperative hours and subsequently at 24 h, and 7, 15, and 30 days postpartum for signs of edema, erythema, discharge, dehiscence, and healing progression. Neonatal viability and survival were monitored throughout the postpartum period.

### 2.7 Statistical Analysis

All statistical analyses were performed using SAS System for Windows (SAS Institute INC., Cary, NC, USA, version 9.3). The data for each response variable were assessed for normality of residuals (Gaussian distribution) and homogeneity of variances using the Guided Data Analysis application. When necessary, variables underwent appropriate transformations. Continuous longitudinal variables with repeated measurements were analysed using linear mixed models for repeated measures. The model included the fixed effects of group, time, and the group × time interaction. When a significant group × time interaction was detected, simple main effects were evaluated to assess group differences at each time point and changes over time within each group. In the absence of a significant interaction, the main effects of group and time were evaluated independently. Pairwise post hoc comparisons were adjusted for multiple testing using the Tukey–Kramer method. Categorical variables were analysed using Fisher's exact test. Categorical data are presented as absolute counts and percentages (*n*/total), together with odds ratios (ORs) and 95% confidence intervals (95% CIs). Continuous data are presented as mean ± standard error of the mean (SEM) together with 95% CIs, except for variables presented as median (interquartile range [IQR]), as appropriate. Statistical significance was set at *p* < 0.05.

## 3 Results

A total of 25 pregnant bitches undergoing elective caesarean section were included in the study, with 15 animals allocated to the Caesarean Group (CG) and 10 animals allocated to the Caesarean plus Ovariohysterectomy Group (COHG). Brachycephalic breeds represented the majority of animals included in both experimental groups, particularly American Bully and French Bulldog bitches. The CG group included American Bully (*n* = 7), French Bulldog (*n* = 5), Golden Retriever (*n* = 2), and Staffordshire Bull Terrier (*n* = 1) bitches, whereas the COHG group included American Bully (*n* = 3), French Bulldog (*n* = 2), Dachshund (*n* = 1), Pit Bull Terrier (*n* = 1), German Spitz (*n* = 1), West Highland White Terrier (*n* = 1), and Rottweiler (*n* = 1) bitches. No significant differences were observed between groups regarding the number of previous parturitions, respiratory rate, heart rate, rectal temperature, or capillary refill time during the preoperative evaluation. However, bitches allocated to the COHG group were significantly older than those in the CG group (3.70 ± 0.42 vs. 2.27 ± 0.23 years, respectively; *p* = 0.0036). Preoperative variables are presented in Table 1. Neonatal Apgar scores did not differ significantly between groups (CG: 4.5 [3.8–6.0]; COHG: 5.31 [4.42–6.0]; *p* = 0.2441). The time to first colostrum intake was also similar between groups, with median values of 144 (85–177) min in the CG and 127 (83–164) min in the COHG (*p* = 0.2784).

Regarding intraoperative findings, no significant differences were observed between groups for surgical time, anaesthetic time, interval between anaesthetic induction and foetal delivery, caesarean section duration, total weight of blood‐contaminated surgical waste, or number of neonates delivered. Maternal vital parameters remained stable throughout the surgical procedure in both groups.

Postoperative and puerperal evaluations demonstrated no major complications associated with either surgical approach. Heart rate, respiratory rate, rectal temperature, and capillary refill time remained within physiological limits throughout the evaluation period. A significant group × time interaction was detected for heart rate; therefore, simple main effects were evaluated. Heart rate differed between groups at 30 days postpartum (*p* = 0.0268), whereas no significant differences were observed at the other assessment times. For respiratory rate and rectal temperature, no significant group × time interactions were detected; however, significant effects of time were observed for respiratory rate (*p* = 0.042) and rectal temperature (*p* < 0.0001), whereas no significant group effects were detected. No significant effects of group or time were observed for capillary refill time. Postoperative physiological variables are summarized in Table 2.

**TABLE 2:** Postoperative maternal physiological variables in bitches undergoing elective caesarean section alone (CG) or elective caesarean section with concomitant ovariohysterectomy (COHG).

A. Main effects of group			
Variable	CG (*n* = 15)	COHG (*n* = 10)	*p*
Respiratory rate (breaths/min)	39.35 ± 1.26	43.26 ± 5.29	0.3331
Rectal temperature (°C)	37.98 ± 0.09	38.04 ± 0.11	0.7688
Capillary refill time (s)	1.92 ± 0.03	1.83 ± 0.06	0.2433

B. Main effects of time						
Variable	0 days	1 days	7 days	15 days	30 days	*p*
Respiratory rate (breaths/min)	32.19 ± 1.96^a^	41.11 ± 5.07^ab^	40.53 ± 2.21^ab^	45.25 ± 6.09^b^	48.29 ± 6.91^b^	0.042
Rectal temperature (°C)	37.13 ± 0.13^a^	38.09 ± 0.08^b^	38.41 ± 0.12^b^	38.27 ± 0.12^b^	38.31 ± 0.13^b^	< 0.0001
Capillary refill time (s)	1.83 ± 0.08	1.86 ± 0.07	1.95 ± 0.05	1.91 ± 0.06	1.88 ± 0.09	0.7847

C. Heart rate: group × time interaction			
Time	CG (*n* = 15)	COHG (*n* = 10)	*p*
0 days	113.20 ± 4.33	126.22 ± 9.48	0.1695
1 day	122.29 ± 4.99	114.50 ± 3.70	0.2934
7 days	125.23 ± 4.52	129.25 ± 7.97	0.6405
15 days	131.69 ± 6.20	118.67 ± 9.02	0.2316
30 days	112.40 ± 5.80	138.67 ± 9.83	0.0268

*Note:* Data are presented as mean ± standard error of the mean (SEM). For respiratory rate, rectal temperature, and capillary refill time, no significant group × time interaction was detected; therefore, the main effects of group and time were evaluated separately. Different superscript letters indicate significant differences between time points within the same variable. For heart rate, a significant group × time interaction was detected; therefore, simple main effects were evaluated to assess group differences at each time point. Pairwise post hoc comparisons were adjusted for multiple testing using the Tukey–Kramer method. Statistical significance was set at *p* < 0.05.

Maternal behaviour evaluation demonstrated adequate maternal care in 86.7% (13/15) of bitches from the CG group and 44.4% (4/9) of bitches from the COHG group. Maternal rejection was observed in 13.3% (2/15) and 55.6% (5/9) of animals from the CG and COHG groups, respectively, although statistical significance was not achieved (*p* = 0.061). One bitch in the COHG group was not included in the maternal behaviour analysis because maternal behaviour could not be assessed. Maternal rejection occurred exclusively during the immediate postoperative period (first postoperative hours and/or within the first 24 h after surgery). During the subsequent evaluations at 7, 15, and 30 days postpartum, all bitches exhibited appropriate maternal care and accepted their litters. Maternal behaviour findings are presented in Table 3.

**TABLE 3:** Comparison of maternal behaviour between experimental groups.

Maternal behaviour	CG (*n* = 15)	COHG (*n* = 9)	OR (95% CI)	*p*
Maternal care	86.7% (13/15)	44.4% (4/9)	7.30 (0.82–105.95)	0.061
Rejection	13.3% (2/15)	55.6% (5/9)	0.14 (0.01–1.22)	0.061

*Note:* Data are presented as *n*/total (%). Odds ratios (OR) and 95% confidence intervals (95% CI) were calculated using the COHG group as the reference category (CG relative to COHG). An OR > 1 indicates higher odds of the outcome in the CG compared to COHG, whereas an OR < 1 indicates lower odds in the CG group.

Abbreviations: CI, confidence interval; OR, odds ratio.

During the first postoperative hours, lactation was observed in 46.7% (7/15) of bitches in the CG group and 66.7% (6/9) in the COHG group, with no significant difference between groups (*p* = 0.423). The remaining bitches were diagnosed with transient hypogalactia and received oxytocin treatment according to the study protocol. When hypogalactia persisted for more than 2 h, supplemental feeding was provided to the neonates according to the standardized protocol of the neonatology team. During the subsequent evaluations at 24 h and 7, 15, and 30 days postpartum, all bitches exhibited adequate milk production and successfully nursed their litters. Lactation findings are summarized in Table 4. No maternal mortality, severe postoperative complications, or neonatal losses associated with the surgical procedures were observed during the puerperal period.

**TABLE 4:** Lactation status during the immediate postoperative period following elective caesarean section.

Lactation	CG (*n* = 15)	COHG (*n* = 9)	OR (95% CI)	*p*
Present	46.7% (7/15)	66.7% (6/9)	0.45 (0.05–3.17)	0.423
Absent	53.3% (8/15)	33.3% (3/9)	2.21 (0.32–19.09)	0.423

*Note:* Data are presented as *n*/total (%). Statistical significance was set at *p* < 0.05. Odds ratios (OR) and 95% confidence intervals (95% CI) were calculated using the COHG group as the reference category (CG relative to COHG). An OR > 1 indicates higher odds of the outcome in the CG compared to COHG, whereas an OR < 1 indicates lower odds in the CG group.

Abbreviations: CI, Confidence interval; OR, odds ratio.

## 4 Discussion

Caesarean section is a common surgical procedure in small animal veterinary obstetrics and represents an important intervention for the management of dystocia and high‐risk pregnancies in bitches (Davidson 2008; De Cramer and Nothling 2020). When future breeding is not intended, ovariohysterectomy may be performed concurrently with caesarean section. However, combining these procedures involves greater surgical manipulation and may increase surgical stress and operative trauma, potentially influencing maternal recovery during the immediate postpartum period. Despite its widespread clinical use, evidence regarding the effects of this combined procedure on perioperative recovery, maternal behaviour, lactation, and early puerperal outcomes remains limited.

The performance of ovariohysterectomy during caesarean section remains controversial because the combined procedure involves additional surgical manipulation and may increase operative trauma, anaesthetic exposure, and the potential for perioperative haemorrhage, with possible consequences for maternal recovery (Davidson 2008; De Cramer and Nothling 2020; Mackeen et al. 2024). Evidence from human obstetrics similarly suggests that more extensive obstetrical procedures may be associated with increased postoperative pain, delayed functional recovery, impaired establishment of breastfeeding, and altered early maternal–infant interaction (Roofthooft et al. 2021; American College of Obstetricians and Gynaecologists [ACOG] 2021). These concerns provide a rationale for evaluating maternal and neonatal outcomes when ovariohysterectomy is performed concurrently with caesarean section.

Evidence regarding the effects of combining caesarean section and ovariohysterectomy on maternal behaviour, lactation, and neonatal outcomes in bitches remains limited and is largely derived from retrospective studies using heterogeneous anaesthetic and surgical protocols (Münnich and Küchenmeister 2014; De Cramer and Nothling 2020; Guest et al. 2023). Guest et al. (2023) evaluated 125 bitches undergoing caesarean section with or without concomitant ovariohysterectomy and did not identify an increased risk of perioperative complications, maternal mortality, or impaired mothering ability associated with the combined procedure. However, direct comparison with the present study should be made cautiously because of differences in study design, sample size, and case selection. In contrast to the larger retrospective population reported by Guest et al. (2023), the present study was prospective, restricted to elective caesarean sections, and included longitudinal assessment of maternal recovery, behaviour, lactation, and puerperal outcomes.

In the present study, concomitant ovariohysterectomy during elective caesarean section was not associated with significant differences in anaesthetic time, surgical time, total weight of blood‐contaminated surgical waste, or postoperative recovery. Because this measure included placentas, blood‐soaked surgical sponges and gauzes, and other disposable materials, it should be interpreted only as an indirect intraoperative measure and not as a direct quantitative assessment of blood loss. Although surgical time was numerically longer in the COHG group, the difference did not reach statistical significance. These findings are consistent with previous retrospective studies reporting no increase in maternal mortality or major perioperative complications when ovariohysterectomy is performed concurrently with caesarean section (Guest et al. 2023; Conze et al. 2020). However, Guest et al. (2023) reported a significantly longer surgical time in bitches undergoing the combined procedure, whereas this difference was not detected in the present study. Differences in study design, case selection, surgical protocols, and sample size may account for this discrepancy. Importantly, the present study evaluated only elective caesarean sections under a standardized anaesthetic and surgical protocol, which may have reduced variability in perioperative management.

Regarding postoperative antimicrobial treatment, all bitches received cephalexin according to the institutional protocol in effect during the study period. Current principles of antimicrobial stewardship emphasize a judicious and evidence‐based approach to antimicrobial use, avoiding routine administration when it is not clinically indicated (Frey et al. 2022; Allerton et al. 2026). Therefore, the antibiotic regimen used in this study reflects the institutional protocol applicable at the time of data collection and should not be interpreted as a recommendation for routine postoperative antimicrobial administration following elective caesarean section. The decision to administer postoperative antimicrobials should be guided by the individual patient's clinical condition and the available evidence.

The longitudinal assessment of maternal physiological variables did not indicate consistent differences between surgical groups. Although a group × time interaction was detected for heart rate, the only between‐group difference occurred at 30 days postpartum, with no differences observed at earlier time points. Respiratory rate and rectal temperature varied over time but did not differ between groups, while capillary refill time remained stable throughout follow‐up. Overall, these findings do not suggest a consistent effect of concomitant ovariohysterectomy on maternal physiological status during the postpartum period.

An important limitation of the present study is the non‐random allocation of bitches to the surgical groups. The decision to perform concomitant ovariohysterectomy was based primarily on the absence of future breeding plans and owner consent, with intraoperative uterine abnormalities additionally influencing the decision in two multiparous bitches. This approach may have introduced selection bias, as the factors influencing group assignment were related to the animals' reproductive characteristics. Accordingly, the groups were not fully comparable at baseline, as reflected by the significantly higher age of bitches in the COHG group. Because age may be associated with reproductive experience and maternal behaviour, it represents a potential confounding factor when interpreting the observed differences in maternal rejection. Given the limited sample size, reliable multivariable adjustment for age was not considered appropriate because of the potential for model instability. Therefore, the difference in maternal age should be considered when interpreting the maternal behavioural findings, and the observed association should not be interpreted as evidence of a causal effect of concomitant ovariohysterectomy.

Another important finding of the present study was the higher frequency of maternal rejection during the immediate postoperative period in bitches undergoing concomitant ovariohysterectomy. Although the difference did not reach statistical significance (13.3% [2/15] in the CG group vs. 55.6% [5/9] in the COHG group; *p* = 0.061), the marked numerical difference warrants consideration as a hypothesis generating finding rather than evidence of a causal effect. Maternal behaviour is fundamental to neonatal survival and early development (Veronesi et al. 2009), and transient disruption of maternal‐neonatal interaction may have clinical relevance during the immediate postpartum period. However, this finding should be interpreted cautiously because of the small sample size, the higher maternal age in the COHG group, and the non‐random allocation of bitches. In addition, although predefined behavioural criteria were applied consistently, assessments were performed by a single investigator without blinding, and no validated quantitative maternal behaviour scale was used; therefore, observer‐related bias and variability in individual behavioural responses cannot be excluded.

The anaesthetic protocol should also be considered when interpreting maternal and neonatal outcomes. Epidural administration of morphine and lidocaine provides effective perioperative analgesia while potentially reducing the requirement for inhalant anaesthetics; however, anaesthetic exposure may still influence maternal recovery and neonatal adaptation. Previous studies evaluating epidural anaesthesia in bitches undergoing elective caesarean section have reported favourable neonatal outcomes, although the effects of anaesthetic protocol on neonatal vitality remain heterogeneous (Antończyk and Ochota 2022; Navarro‐Altuna et al. 2025). Propofol and isoflurane can cross the placenta, and neonatal effects may depend on drug exposure, anaesthetic duration, and the interval between induction and foetal extraction. Prolonged exposure to isoflurane has been associated with lower neonatal Apgar scores and alterations in umbilical blood gas parameters when foetal extraction is delayed after induction (Antończyk et al. 2023). In the present study, the interval between anaesthetic induction and extraction of the first neonate was recorded, and neonatal viability was objectively assessed using the Apgar score. However, umbilical blood gas analysis and other biochemical measures of neonatal adaptation were not performed, and subtle effects of anaesthetic exposure cannot therefore be completely excluded.

Postoperative analgesic treatment may also have influenced maternal behaviour during the immediate postpartum period. All bitches received the same standardized analgesic regimen consisting of dipyrone and tramadol, reducing the likelihood that differential analgesic exposure accounted for the between‐group difference in maternal rejection. Dipyrone has been reported to provide adequate postoperative analgesia in bitches without clinically relevant adverse effects, although available evidence derives from ovariohysterectomy rather than caesarean section (Imagawa et al. 2011). Tramadol has been evaluated in bitches after caesarean section, and both the drug and its active metabolite have been detected in colostrum following intravenous administration (Somrup et al. 2018). Nevertheless, individual differences in pain, sedation, and behavioural responses could not be excluded and were not systematically quantified in the present study. Therefore, a potential contribution of postoperative pain or drug effects to the transient maternal rejection observed immediately after surgery cannot be completely ruled out.

Maternal behaviour in bitches is influenced by complex endocrine and physiological adaptations occurring around parturition, particularly involving hormonal and sensory stimuli associated with maternal‐neonatal interaction (Concannon 2011; Guardini et al. 2015). Elective caesarean section performed before the onset of spontaneous labour may interfere with the normal neuroendocrine events associated with parturition, including the release of oxytocin and prolactin, which are involved in maternal behaviour, lactation, and postpartum uterine function (Fusi and Veronesi 2022). Although progesterone concentrations have already declined at term, the addition of ovariohysterectomy to caesarean section increases the extent of surgical manipulation and could potentially influence postpartum maternal adaptations. In the present study, the higher frequency of maternal rejection in the COHG group raises the possibility that concomitant ovariohysterectomy may influence maternal behaviour during the immediate postpartum period. However, this interpretation remains speculative because hormonal variables were not evaluated. Interestingly, lactation did not differ significantly between groups despite the higher frequency of maternal rejection, suggesting that transient alterations in maternal behaviour may occur without an evident impairment of milk production. The mechanisms underlying this apparent dissociation remain unclear and warrant further investigation. Nevertheless, although a standardized manual milking protocol was used, individual variation in milk ejection may have influenced the volume obtained. This possibility should be considered when interpreting the results.

The increased frequency of maternal rejection may also have been influenced by factors unrelated to hormonal alterations, including postoperative pain, residual effects of anaesthetic or analgesic drugs, surgical and environmental stress, individual behavioural variation, breed‐related temperament, parity, and previous maternal experience (Santos et al. 2020; Uchańska et al. 2022). Although all bitches received the same standardized postoperative analgesic regimen and no anxiolytic or tranquillising drugs were administered, individual differences in pain and behavioural responses could not be excluded and were not systematically quantified. Likewise, differences in owner interaction or the home environment during subsequent postpartum assessments may have influenced maternal behaviour (Santos et al. 2020). These factors therefore represent potential confounders and further limit the interpretation of the observed association between concomitant ovariohysterectomy and maternal rejection.

Importantly, maternal rejection was observed exclusively during the immediate postoperative period. At 7, 15, and 30 days postpartum, all bitches exhibited appropriate maternal care and accepted their litters. This temporal pattern suggests that the observed behavioural alteration, when present, was transient rather than a persistent impairment of maternal care. Nevertheless, given the small sample size, the higher maternal age in the COHG group, the non‐random allocation of bitches, and the lack of blinded behavioural assessment using a validated quantitative scale, larger prospective studies are needed to determine whether concomitant ovariohysterectomy has a clinically relevant effect on maternal behaviour.

Neonatal outcomes were primarily evaluated in terms of immediate vitality and survival. Immediate neonatal care was standardized and performed by a dedicated team, and neonatal vitality was objectively assessed using the Apgar score. Previous studies have demonstrated that foetal maturity, prompt neonatal assistance, and adequate maternal care are important determinants of neonatal survival after caesarean section in bitches (Groppetti et al. 2010; Veronesi et al. 2009). The standardized neonatal management used in the present study may therefore have reduced the influence of differences in immediate perioperative care on the comparison between groups. Nevertheless, because neonatal growth and development were not evaluated longitudinally, potential effects of early maternal behavioural differences on subsequent neonatal outcomes could not be assessed.

The present study has some limitations that should be considered when interpreting the findings. The relatively small sample size may have limited the statistical power to detect differences, particularly categorical outcomes such as maternal rejection and lactation; therefore, clinically relevant differences between groups cannot be excluded. The non‐random allocation of bitches and the baseline difference in maternal age may have introduced selection bias and confounding. In addition, postoperative haematological parameters were not reassessed, precluding evaluation of potential changes in packed cell volume, haematocrit, red blood cell count, and related parameters associated with perioperative blood loss. Hormonal evaluation was also not performed, limiting the interpretation of potential endocrine mechanisms underlying postpartum maternal responses. Maternal behaviour was assessed by a single investigator without blinding or a validated quantitative behavioural scale, and breed heterogeneity may also have contributed to variability in obstetrical and maternal behavioural characteristics. Finally, neonatal outcomes were primarily evaluated during the immediate postpartum period, precluding assessment of potential longer‐term effects on neonatal growth and development.

Despite these limitations, this is one of the few prospective clinical studies evaluating puerperal outcomes in bitches undergoing concomitant ovariohysterectomy during caesarean section. Under the standardized surgical and anaesthetic conditions used in the present study, concomitant ovariohysterectomy was not associated with significant differences in perioperative recovery, lactation, neonatal viability, or longer‐term maternal care. However, the small sample size and non‐random allocation limit the strength of these conclusions, particularly regarding the observed difference in maternal rejection. Larger prospective studies with standardized and objective assessments of maternal behaviour, endocrine responses, haematological variables, and neonatal development are warranted to determine whether the combined procedure has clinically relevant effects.

## 5 Conclusions

In bitches undergoing elective caesarean section, concomitant ovariohysterectomy was not associated with significant differences in perioperative recovery, maternal physiological variables, lactation, neonatal viability, or subsequent maternal care compared with caesarean section alone. However, a higher frequency of transient maternal rejection was observed during the immediate postoperative period in bitches undergoing concomitant ovariohysterectomy, although this difference did not reach statistical significance. Given the small sample size, non‐random allocation, and baseline differences between groups, these findings should be interpreted cautiously. Under the standardized surgical and anaesthetic conditions used in the present study, concomitant ovariohysterectomy was not associated with clinically evident adverse perioperative or neonatal outcomes.

## Author Contributions

Juliana Dantas Bento conceived and designed the study, collected, analysed and interpreted the data, and participated in manuscript writing. Mariana Semião Francisco Talib collected the data, analysed and interpreted the data. Marcelo Faustino collected the data, analysed and interpreted the data. Álvaro de Miranda Alves analysed and interpreted the data and critically revised the manuscript. Gisele Almeida Lima da Veiga designed the study, collected, analysed and interpreted the data, participated in manuscript writing, critically revised the manuscript, and agreed to be accountable for all aspects of the work. All authors approved the final version of the manuscript.

## Funding

This study was supported by the University of São Paulo through its Unified Scholarship Program (Programa Unificado de Bolsas ‐ PUB), grant number 2024‐6022.

## Conflicts of Interest

The authors declare no conflicts of interest.

## Data Availability

The data that support the findings of this study are available on request from the corresponding author. The data are not publicly available due to privacy or ethical restrictions.
